# Effects of Freeze–Thaw Cycles and Basalt Fiber Reinforcement on the Mechanical Properties and Constitutive Model of Lunar Regolith Simulant Geopolymer Under In Situ Construction Environments

**DOI:** 10.3390/polym18101169

**Published:** 2026-05-09

**Authors:** Jianghuai Zhan, Xuanyi Xue, Haolan Yi, Fei Wang, Shuai Li, Jianmin Hua

**Affiliations:** 1 School of Civil Engineering, Chongqing University, Chongqing 400045, China; 2 State Key Laboratory of Safety and Resilience of Civil Engineering in Mountain Area, Chongqing 400045, China; 3 Department of Civil Engineering, The University of Hong Kong, Pokfulam Road, Hong Kong, China

**Keywords:** LRS geopolymer, basalt fiber, freeze–thaw cycle, compressive strength, flexural strength, stress–strain constitutive model

## Abstract

This study investigated the effects of freeze–thaw cycles on unreinforced and basalt fiber-reinforced lunar regolith simulant (LRS) geopolymer. Specimens were subjected to 0, 3, 6, and 10 freeze–thaw cycles. Compressive strength, flexural strength, elastic modulus, peak strain, and failure mode were measured. Damage degree and gain ratio were used to evaluate fiber reinforcement. Results showed that the unreinforced LRS geopolymer exhibited considerable fluctuation in compressive strength during freeze–thaw cycles. Its compressive strength first increased, then decreased; its flexural strength continuously declined; and its elastic modulus and peak strain showed opposite trends, with typical brittle failure. In contrast, basalt fiber-reinforced LRS geopolymer demonstrated superior frost resistance. Its compressive strength increased continuously with freeze–thaw cycles, reaching 23.5% after 10 cycles. Its flexural strength decreased but stabilized, with a damage level of only 16.0% after 10 cycles, significantly lower than that of the unreinforced group (26.1%). Its elastic modulus increased continuously while peak strain decreased gradually, with failure exhibiting some ductile characteristics. Gain ratio analysis showed compressive and flexural strength gain ratios of 1.92 and 1.69, respectively, after 10 cycles, indicating significant reinforcement. Among three classical constitutive models (Guo Zhenhai, Saenz L.P., and Carreira D.J.), the Guo Zhenhai model provided the best fit for stress–strain curves of both geopolymer types under all freeze–thaw conditions, making it the recommended constitutive model. This study provides theoretical support for LRS geopolymer applications in extreme environments such as the lunar surface.

## 1. Introduction

Establishing a lunar base is a critical objective in human near-space exploration. Lunar regolith is a layer of loose granular material covering the lunar bedrock, primarily formed by long-term processes such as meteorite impact fragmentation, cosmic ray bombardment, and extreme temperature variations. The utilization of in situ lunar regolith to produce construction materials is considered a key pathway to achieving this goal. As the most abundant resource on the lunar surface, lunar regolith has a complex and diverse mineral composition, mainly including primary rock fragments (such as basalt and anorthosite), mineral fragments (such as pyroxene, olivine, and plagioclase), glassy particles (including volcanic glass and impact glass), and agglutinates. Its geological characteristics are similar to those of terrestrial volcanic ash, demonstrating the potential to produce geopolymers through alkali-activated reactions [1]. However, due to the difficulty in obtaining genuine lunar regolith and its high cost, conducting systematic research directly with authentic lunar samples presents considerable limitations [2]. Consequently, the preparation of geopolymer construction materials using lunar regolith simulant (LRS) has emerged as a prominent research focus within the international academic community. Related investigations have encompassed multiple aspects, including material mix proportion optimization, reaction mechanism analysis, and mechanical property characterization, thereby establishing a significant theoretical foundation for in situ lunar construction [3].

As a brittle material, LRS geopolymer exhibits inherent defects such as low tensile strength and poor toughness, rendering it susceptible to brittle fracture without significant warning under flexural or impact loads, thereby posing a threat to the structural safety of lunar bases [4]. Therefore, enhancing its toughness and crack resistance is crucial for promoting its engineering application. Research has demonstrated that fiber incorporation is an effective approach to improving the mechanical performance of brittle materials [5]. Shi et al. [6] successfully fabricated LRS fibers using an automatic wire-drawing device under vacuum conditions. They observed that a vacuum environment facilitated the formation of continuous fibers, achieving a tensile strength of 1205 MPa—37% higher than that of fibers prepared in air. The underlying mechanism was attributed to the anoxic conditions, which increased the Fe^2+^/ΣFe ratio and reduced the content of non-bridging oxygen, thereby optimizing the surface structure and enhancing acid resistance. Zhan et al. [4] investigated the effect of basalt fiber content (0–0.4%) on the mechanical properties of LRS geopolymers. The results indicated that under strong alkali activation and curing at 20 °C for 7 d, the optimal ductility was achieved with a fiber content of 0.3%. Under strong alkali activation and curing at 80 °C for 1 d, the highest strength was obtained at 0.2% fiber content, although the failure mode remained brittle. Under weak alkali activation and curing at 80 °C for 7 days, a fiber content of 0.1% yielded the best overall performance, with a flexural strength of 3.88 MPa. Meng et al. [7] examined the influence of polypropylene fiber length (3, 6, and 9 mm) and content (0–0.4 wt.%) on LRS geopolymers. The findings revealed that fiber incorporation reduced the fluidity of the paste, with 9 mm fibers exhibiting the most pronounced effect. The optimal reinforcement was observed at a fiber length of 6 mm and a content of 0.4 wt.%, where the compressive strength, flexural strength, and flexural toughness increased by 54.6%, 117.7%, and 90%, respectively. The toughening efficiency followed the order of 6 mm > 9 mm > 3 mm. Li et al. [8] compared the reinforcing effects of basalt fiber (BF), polyvinyl alcohol fiber (PVA), and polypropylene fiber (PP) on LRS geopolymers. The results demonstrated that PP exhibited the best performance, with a fiber content of 0.4 wt.% increasing compressive strength, flexural strength, and mid-span deflection by 54.5%, 117.7%, and 90%, respectively. The toughening effectiveness ranked as PP > PVA > BF. Current research on fiber-reinforced LRS geopolymers has predominantly focused on PP and PVA fibers, while systematic investigations on BF remain limited. Lunar regolith is rich in silicate minerals, compositionally similar to basalt, enabling the in situ preparation of basalt fibers through melt-drawing technology. These fibers possess excellent mechanical properties and chemical stability, making them an ideal candidate for reinforcing LRS geopolymers. Therefore, basalt fiber was selected as the primary reinforcing phase in this study.

It should be noted that the lunar regolith is generally extremely dry with very low water content. According to analyses of Apollo mission samples, the water content in lunar highland anorthosite is approximately 6 ppm, and the initial water content of the lunar magma ocean is about 320 ppm [9]; the LCROSS mission detected the presence of water ice in the permanently shadowed regions of the lunar poles, with a content of approximately 5.6 wt% [10]. Therefore, in the equatorial and mid-latitude regions of the Moon, the classical freeze–thaw mechanism based on pore-water freezing and associated expansion stress is not directly applicable. However, in the permanently shadowed regions of the lunar poles, where temperatures can drop below −230 °C, the possibility of water-ice enrichment exists, and freeze–thaw effects may become a potential factor affecting material durability. Furthermore, even under anhydrous conditions, extreme temperature variations (with a temperature difference exceeding 300 °C) [11] themselves can induce significant thermal stress damage due to differences in the coefficients of thermal expansion of minerals, ultimately affecting the mechanical properties and long-term durability of materials [12,13]. For LRS geopolymers intended for in situ lunar construction, the freeze–thaw effect is a critical environmental factor to consider. Wang et al. [14] prepared geopolymers using volcanic ash as raw material and NaOH as an activator, and evaluated their performance under vacuum and freeze–thaw conditions. The results showed that after 24 h of vacuum exposure and 30 freeze–thaw cycles in liquid nitrogen, the material experienced a slight decrease in strength, yet still met the requirements for mechanical performance. Pilehvar et al. [15] incorporated urea into DNA-1 lunar regolith simulant geopolymers, cured the specimens at 80 °C for 6 h, and then subjected them to freeze–thaw cycles ranging from −80 °C to 80 °C. The findings indicated that urea enhanced the strength of the geopolymers after freeze–thaw exposure. Further investigation revealed that while simulated lunar temperature cycling increased compressive strength, a vacuum environment had a detrimental effect on mechanical performance. Tabuchi et al. [16] investigated the freeze–thaw permeability of LHS-1 lunar highland simulant. The results demonstrated that after a single freeze–thaw cycle, the permeability increased by up to 6.4 times compared to that before cycling, with lower packing densities (ranging from 0.49 to 0.60) exhibiting more significant increases. Prior to freeze–thaw cycling, the horizontal permeability at a packing density of 0.6 was 1.6 × 10^−13^ m^2^. The vertical permeability at the lunar surface was estimated to be 6 × 10^−13^ to 2 × 10^−12^ m^2^, and at a depth of 5 m, it ranged from 7 × 10^−14^ to 3 × 10^−13^ m^2^. The increase in permeability was attributed to particle rearrangement induced by the freezing of pore water. This study provided key parameters for understanding water molecule migration and resource utilization on the lunar surface. Liu et al. [17] reported that high-temperature-to-ultra-low-temperature cycling affected both the compressive and flexural strengths of LRS materials solidified with a cementitious binder. This degradation was attributed to the combined effects of thermal stress arising from differences in coefficients of thermal expansion and pore frost heave forces generated during the low-temperature phase. Some scholars have studied the performance degradation of structural materials under extreme temperature variations [18,19,20,21]. These mechanisms led to the coalescence and expansion of small-scale pores, resulting in pore coarsening, interfacial debonding, or structural damage, thereby reducing the mechanical properties of the material.

In summary, current research on LRS geopolymers has primarily focused on aspects such as mix proportion optimization, fiber reinforcement effects, and characterization of conventional mechanical properties. However, investigations into the durability of these materials under simulated extreme lunar temperature environments remain insufficient, particularly regarding the mechanical evolution laws and constitutive relationships of basalt fiber-reinforced systems subjected to freeze–thaw cycles. To address this gap, this study systematically conducted mechanical performance tests on basalt fiber-reinforced LRS geopolymers under freeze–thaw cycling conditions, revealing the evolution patterns of compressive strength, flexural strength, stress–strain behavior, and failure modes of the composite material under different numbers of freeze–thaw cycles, and established a constitutive model applicable to freeze–thaw environments. The core innovations of this study are as follows: it focuses on the freeze–thaw durability under extreme lunar temperature environments, filling a gap in the environmental adaptability research of in situ lunar construction materials; it systematically reveals the reinforcement and failure mechanisms of basalt fibers under freeze–thaw cycles, highlighting their unique advantages over organic fibers in terms of in situ lunar manufacturability and chemical stability; and it establishes, for the first time, a specialized constitutive model for freeze–thaw cycling conditions, providing a continuous and quantitative description tool for the mechanical behavior of the material under alternating temperature fields.

## 2. Materials and Experiment Methods

### 2.1. Materials

**Figure 1:**
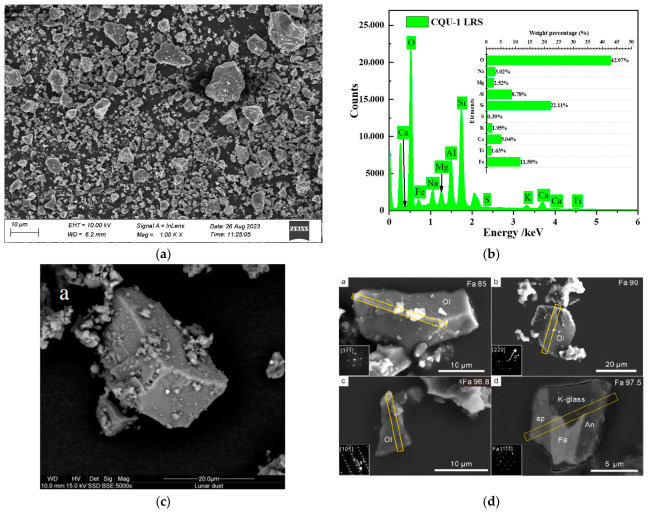
SEM comparison of CQU-1 LRS and real lunar regoliths: (**a**) CQU-1 LRS; (**b**) EDS image of CQU-1 LRS; (**c**) Apollo 16 lunar regolith [27]; (**d**) Chang’E-5 lunar regolith [22]. Note: In subfigure d, a, b, c, and d respectively represent the SEM morphology and diameter of different lunar regolith particles from Chang’E-5 lunar regolith.

**Figure 2:**
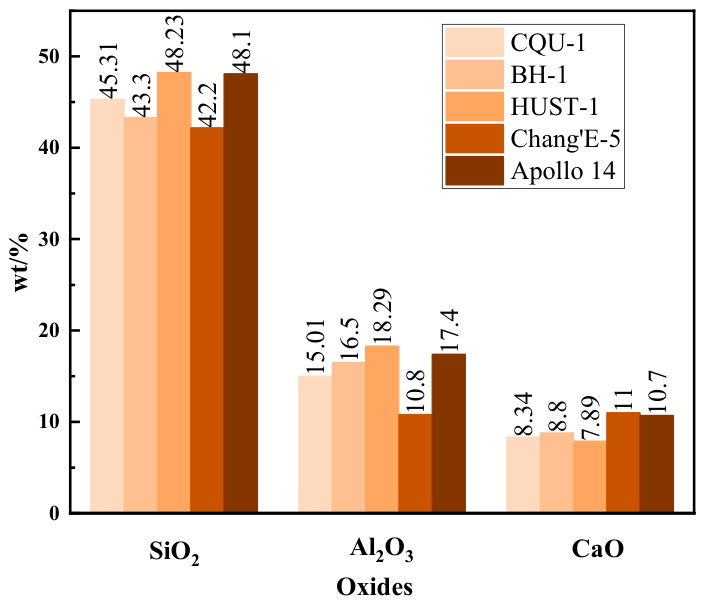
Chemical composition of real lunar regolith and LRSs.

The CQU-1 LRS was used in this study. The same material was also employed in Ref. [3]. Based on the current investigations, X-ray fluorescence (XRF) analysis was employed to systematically compare the chemical composition characteristics among the Chang’E-5 lunar regolith [22], Apollo 14 lunar regolith [23], and the LRSs, including BH-1 from Beihang University [24], HUST-1 from Huazhong University of Science and Technology [25], and the CQU-1 used in this study. As shown in Table 1, XRF analysis revealed that the major chemical components of the LRSs, Chang’e-5 lunar regolith [22], and Apollo 14 lunar regolith [23] include SiO_2_, TiO_2_, Al_2_O_3_, Fe_2_O_3_, CaO, MgO, Na_2_O, K_2_O, and P_2_O_5_. The EDS spectrum of CQU-1 LRS raw materials (Figure 1) demonstrated that it primarily consists of Na, Si, Al, Fe, Ca, Mg, K, Ti, and O elements. The content ratios of key components such as SiO_2_, Al_2_O_3_, and CaO in real lunar regolith and LRS are shown in Figure 2. The formation of geopolymers fundamentally relies on the dissolution and polymerization of active silicon-aluminum components in an alkaline environment. Given that LRS exhibits a highly similar silicon-aluminum elemental composition to real lunar regolith, it possesses the material basis for substituting real lunar regolith in geopolymer preparation. SEM images further revealed that the CQU-1 LRS is highly similar to real lunar regolith in terms of particle morphology and surface structure characteristics. The chemical composition is consistent with the typical elemental profile of real lunar regolith. Based on this similarity analysis, the present study employed the CQU-1 LRS as a substitute for real lunar regolith in subsequent experiments.

**Table 1:** Chemical component of real lunar regolith and LRSs (wt%).

Oxides	SiO_2_	TiO_2_	Al_2_O_3_	Fe_2_O_3_	CaO	MgO	Na_2_O	K_2_O	P_2_O_5_
CQU-1 [3]	45.31	2.80	15.01	15.67	8.34	3.41	4.50	3.33	0.65
BH-1 [24]	43.30	2.90	16.50	16.70	8.80	3.00	3.80	3.30	0.70
HUST-1 [25]	48.23	2.96	18.29	11.19	7.89	4.41	3.70	2.15	0.50
Chang’E-5 [22]	42.20	5.00	10.80	22.50	11.00	6.48	0.26	0.19	0.23
Apollo 14 [23]	48.10	1.70	17.40	10.40	10.70	9.40	0.70	0.55	0.51

The alkaline activator employed in this study was formulated as a composite solution comprising sodium silicate, sodium hydroxide, and deionized water. The sodium silicate component exhibited a modulus of 3.3, with precise chemical compositions of 26.5 wt% SiO_2_ and 8.3 wt% Na_2_O. High-purity sodium hydroxide flakes (≥99%) were incorporated to precisely regulate the silicate modulus, while deionized water functioned as the solvent medium to ensure chemical purity throughout the experimental process.

The basalt fibers used in this study were produced by Anjie Company, China. Chopped basalt fibers were used at a dosage of 0.1% by mass of the binder. The fiber specifications are presented in Table 2. The fibers had a monofilament diameter of 10 μm, a density of 2.63–2.65 g/cm^3^, an elastic modulus of 91–110 GPa, and a tensile strength of 3000–4800 MPa. Each fiber bundle consisted of 1200 monofilaments. These parameters are identical to those of the basalt fibers selected in a previous study [26].

### 2.2. Experimental Variable Design

Lunar lava tubes are special underground caves formed by volcanic eruptions and are considered ideal natural shelters and scientific laboratories for constructing lunar bases. Feng et al. [28] conducted temperature measurements in both illuminated and shadowed areas of a collapsed lava tube and observed that while the lunar surface temperature ranged from −170 °C to 110 °C, the shadowed areas without direct sunlight maintained a temperature between −20 °C and 30 °C. Due to the advantages of natural shelter and relatively minor temperature variations, lunar lava tubes are regarded as promising sites for lunar base construction. Therefore, in this experiment, the freeze–thaw cycle temperature was set between −20 °C and 30 °C to simulate the conditions within shadowed lunar lava tubes without direct sunlight.

The key variables in this study were set as the incorporation of basalt fiber (with or without) and the number of freeze–thaw cycles (0, 3, 6, and 10). Under weak alkali excitation, basalt fibers with a length of 15 mm were incorporated at a content of 0.1% to reinforce the specimens. The specimens were cured at 80 °C for one day to prepare the LRS geopolymer and the basalt fiber-reinforced LRS geopolymer, respectively. After curing was completed and the specimens returned to room temperature, they were subjected to temperature cycles ranging from −20 °C to 30 °C. Each cycle consisted of placing the specimens at −20 °C for 11 h, followed by rewarming and subsequent exposure to 30 °C for 11 h. After subjecting the LRS geopolymer and basalt fiber-reinforced LRS geopolymer to 0, 3, 6, and 10 freeze–thaw cycles, a comprehensive analysis of the mechanical properties of the geopolymers was conducted. The aim was to investigate the influence of freeze–thaw cycles on the mechanical properties of LRS geopolymer and the effect of basalt fiber incorporation on the durability of LRS geopolymer, and to establish a constitutive model for the geopolymer after freeze–thaw cycles. To enhance the reliability of the experimental results, three parallel specimens were designed for each group. A total of 24 compressive specimens and 24 flexural specimens were prepared in this chapter, amounting to 48 specimens in total.

It should be noted that the lunar surface environment is extremely dry and lacks free water, so the classical mechanism of freeze–thaw damage based on pore-water freezing and associated expansion stress is not directly applicable to the in situ lunar environment. The freeze–thaw test conducted in this study under water-saturated conditions is not intended to directly simulate the water environment on the Moon, but rather serves as an accelerated testing method. By utilizing free water as an explicit damage factor, this approach accelerates the deterioration process of the material under extreme temperature differences in a controllable manner, thereby enabling a systematic evaluation of the mechanical response of LRS geopolymers under alternating temperature conditions and the reinforcing effect of basalt fibers. Therefore, the results of this study provide a valuable reference for the performance assessment of in situ lunar construction materials. However, when extrapolating these findings to the actual lunar environment, further consideration should be given to the coupled effects of vacuum, lack of water, and reduced gravity.

The basalt fiber content (0.1%) and length (15 mm) were determined based on preliminary mix optimization tests. The results showed that when the fiber content exceeded 0.1%, the fluidity of the fresh mixture decreased significantly, and a fiber length of 15 mm provided the best reinforcing effect while maintaining adequate workability. This finding is consistent with the study by Zhan et al. [4], which also identified 0.1% as the optimal basalt fiber content for LRS geopolymers. The curing condition (80 °C for 24 h) was adopted following the study by Lu et al. [29], which demonstrated that this regime enables LRS geopolymers to achieve more than 80% of their ultimate strength while avoiding excessive shrinkage and microcracking caused by higher temperatures or prolonged curing. The maximum number of freeze–thaw cycles (10 cycles) was set based on preliminary tests, which revealed that unreinforced specimens developed significant macroscopic cracks and exhibited a strength reduction exceeding 30% after 10 cycles. Further cycling contributed little to elucidating the fiber reinforcement mechanism, and 10 cycles were sufficient to distinguish the performance evolution between the unreinforced and fiber-reinforced groups. This setting is comparable to those adopted by Tabuchi et al. [16] and Liu et al. [17] in their studies on LRS materials.

### 2.3. Geopolymer Specimen Preparation

This study prepared LRS geopolymers in accordance with GB/T 17671-2021 [30] and BS EN 196-1: 2016 [31]. A weak alkali-activated system was adopted in this experiment, using pure sodium silicate solution as the alkali activator, with a modulus of 3.3 and an alkali content (calculated as Na_2_O) of 8.3%. The water-binder ratio was controlled at 0.456. According to the designed mix proportions, the LRS raw material was weighed into a stainless steel mixing bowl and mixed using a JJ-5 planetary cement mortar mixer (Nanjing Taiste Instrument Equipment Co., Ltd., Nanjing, China). After adding the specified mass of alkali activator, the mixture was first stirred at a low speed (140 ± 10 r/min) for 2 min, followed by high-speed mixing (285 ± 10 r/min) for 3 min. The resulting paste was cast into 40 mm cube molds and 40 mm × 40 mm × 160 mm prism molds, placed on a vibrating table to eliminate air bubbles, smoothed, and then sealed with polyethylene film to prevent moisture evaporation. The specimens were cured in an oven at 80 ± 1 °C for 24 h before demolding. After demolding, the freeze–thaw cycle experiments were conducted. Detailed mix proportion parameters are presented in Table 3.

### 2.4. Mechanical Test Method

The compressive performance was evaluated using an MTS CMT5105 electronic universal testing machine (MTS Industrial Systems (China) Co., Ltd., Shenzhen, China). The displacement-controlled loading protocol was applied at a constant rate of 0.15 mm/min until specimen failure occurred, accompanied by a significant reduction in load-bearing capacity. The data for each test group represented the average value obtained from three parallel specimens. During testing, the system automatically recorded mechanical parameters at a frequency of 30 Hz. The load-deformation curves generated post-test provided critical insights into the material’s mechanical response characteristics. In this study, mechanical properties, including compressive strength, flexural strength, elastic modulus, and peak strain, were obtained based on the test results of three parallel specimens per group. The average value was taken as the final representative value, and error bars were added to the corresponding figures to indicate data dispersion. The failure mode images were selected from representative specimens exhibiting typical failure characteristics in each group. It should be noted that the stress–strain curves presented in this study are not the arithmetic mean of three curves. Instead, the curve closest to the average response was chosen as the representative curve for each group. In this study, 48 specimens were prepared and tested: 24 for compressive strength tests and 24 for flexural strength tests.

The flexural strength of the geopolymer specimens was tested in accordance with GB/T 17671-2021 [30], using prismatic specimens measuring 40 mm × 40 mm × 160 mm. The test was performed using a universal testing machine with a three-point bending configuration. The span length between the two support rollers was 100 mm. The loading roller was positioned at the mid-span of the specimen, and the load was applied at a constant rate of 50 N/s until failure. The flexural strength *R*_f_ (MPa) was calculated using the following formula:

(1) $$R_{f}=\frac{1.5\times F_{f}\times L}{b^{3}}$$

where *F_f_* is the maximum applied load at failure (N); *L* is the span length (100 mm); *b* is the side length of the square cross-section of the specimen (40 mm).

**Figure 3:**
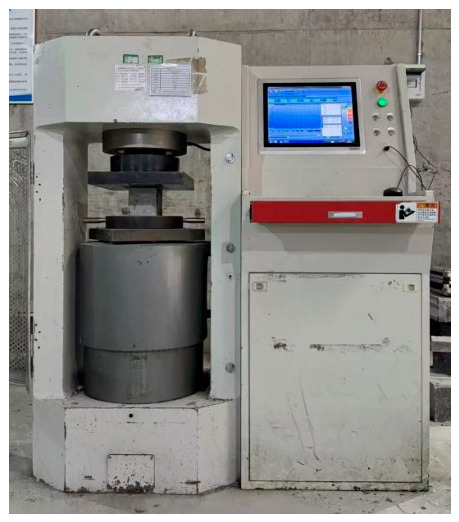
Test setup for stress–strain curve measurement.

The experimental setup for compressive property and stress–strain curve testing in this study utilized an MTS CMT5105 electronic universal testing machine, as illustrated in Figure 3. During testing, 50 mm adhesive-based strain gauges were securely and uniformly attached to the mid-section of all four lateral surfaces of the specimen in both longitudinal and transverse orientations. These gauges were connected to a TDS-150 static data acquisition system to monitor specimen deformation in real time. The specimen was then positioned on the lower loading platform and subjected to displacement-controlled loading at a rate of 0.15 mm/min until failure occurred and the load-bearing capacity dropped significantly, with load data being recorded by a pressure sensor. To minimize experimental error, an additional displacement transducer was installed at the lower loading end of the testing machine to record the displacement-time history of the specimen via digital acquisition. Compressive strength values were determined by averaging the results from three parallel specimens, while the stress–strain curve selected for analysis was the one closest to the average response.

In this study, the 50 mm strain gauge length was selected because the maximum particle size of the LRS used is less than 2 mm. The 50 mm gauge length is approximately 25 times the maximum particle size, well exceeding the general requirement that the gauge length should be at least three times the maximum aggregate size, thus ensuring the representativeness of the measured deformation. Therefore, the 50 mm gauge length is appropriate for this study.

**Figure 4:**
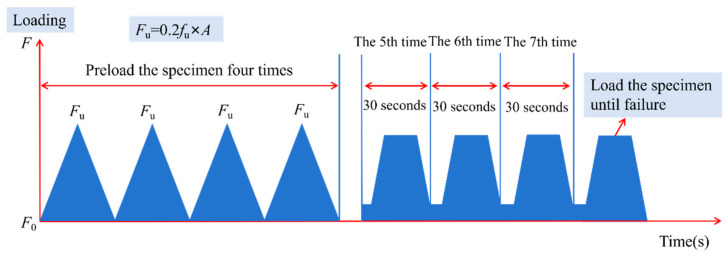
Schematic of the elastic modulus test loading procedure.

The elastic modulus test was conducted in accordance with the Chinese standard GB/T 50081-2019 [32]. The elastic modulus test setup is shown in Figure 4. For this test, two opposing surfaces of the specimen were ground flat, and the cross-sectional area A of the specimen was measured. A 300 kN microcomputer-controlled electro-hydraulic servo universal testing machine was used to apply cyclic loading at a rate of 0.1 kN/s. The upper load limit was set to 0.2 *f*_u_ (where *f*_u_ is the ultimate compressive strength of the specimen). The procedure included three initial preloading cycles followed by three formal preloading cycles, each with a 30-s load-holding period. The test was terminated when the deformation difference between any two consecutive loading cycles did not exceed 0.2‰ of the measurement gauge length.

The elastic modulus of the specimen was calculated using the following Equation:

(2) $$E_{c}=\frac{F_{a}-F_{0}}{A}\times \frac{l}{\Delta l}$$

where *E*_c_ is the elastic modulus of the specimen (MPa); *A* is the cross-sectional area of the specimen (mm^2^); *F*_a_ = 0.2 *f*_u_, *f*_u_ is the ultimate compressive strength (N). *F*_0_ = 0.1 *f*_u_, Δ*l* is the deformation of the specimen when loaded to *F*_a_; *l* is the height of the specimen (mm).

Regarding the reason for selecting 0.2 *f*_u_ instead of 0.5 *f*_u_ as the upper load value: the LRS geopolymer used in this study is highly brittle, with a relatively short linear elastic region in its stress–strain curve. To ensure that the elastic modulus test was conducted strictly within the linear elastic range and to avoid the influence of plastic deformation on the results, we referred to the testing recommendations for brittle materials (e.g., rock, ceramic) and set the upper load to 0.2 *f*_u_. Furthermore, Xiong et al. [33] also adopted 0.2 *f*_u_ as the upper load value for elastic modulus testing in their study on LRS geopolymer, and our testing method is consistent with theirs.

The cyclic loading was applied between 0 MPa (*F*_0_) and a predefined upper stress level, with each peak load held constant for 30 s. A total of five such loading cycles were conducted, with data from the fourth cycle (as indicated) used for analysis.

## 3. Results and Discussions

### 3.1. Mechanical Property

#### 3.1.1. Compressive Strength and Failure Mode

**Figure 5:**
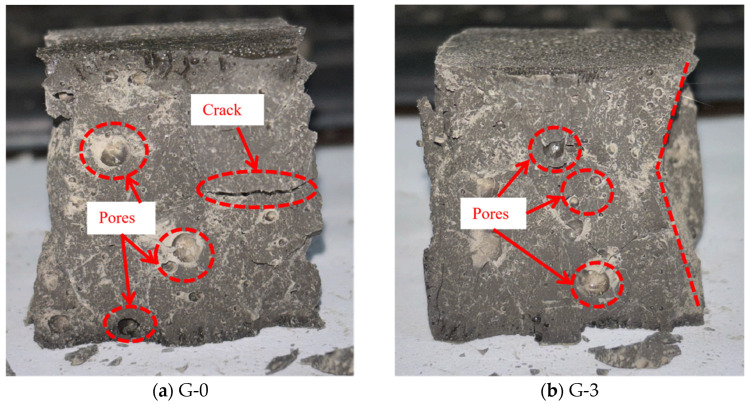

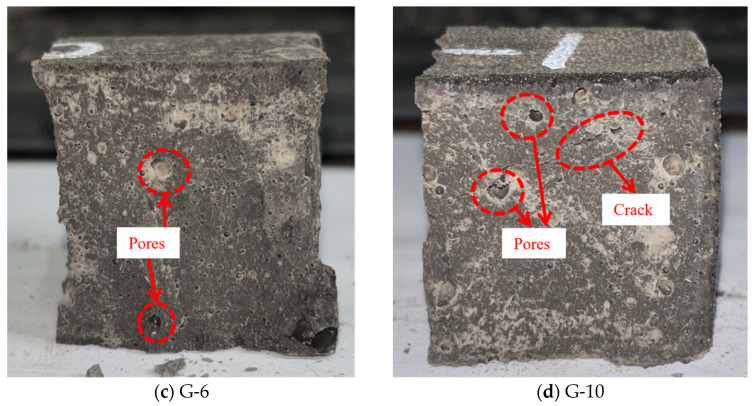
Failure modes of unreinforced LRS geopolymer compressive specimens after different numbers of freeze–thaw cycles.

The failure modes of unreinforced LRS geopolymer specimens after different numbers of freeze–thaw cycles are shown in Figure 5. The control group (0 cycles) exhibited typical brittle failure: a crisp sound was emitted during loading, followed by spalling and rapid instability of the specimen. After 3 and 6 freeze–thaw cycles, the failure patterns were similar to those of the control group, still characterized by brittle fracture. When the number of freeze–thaw cycles reached 10, the specimens lost their load-bearing capacity instantaneously upon emitting a crisp sound, exhibiting a more sudden failure. Overall, as the number of freeze–thaw cycles increased, the brittleness of the geopolymer intensified while its ductility diminished.

**Figure 6:**
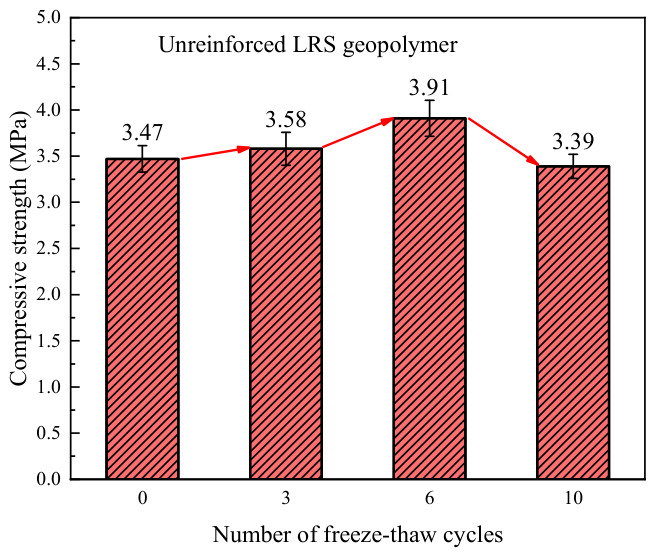
Compressive strength of unreinforced LRS geopolymers after different numbers of freeze–thaw cycles.

The compressive strength of unreinforced LRS geopolymer after different numbers of freeze–thaw cycles is shown in Figure 6. Overall, the compressive strength ranged from 3.39 to 3.47 MPa, exhibiting an initial increase followed by a decrease as the number of freeze–thaw cycles increased. Compared with the control group, the strength changed by +3.2%, +12.7%, and −2.3% after 3, 6, and 10 freeze–thaw cycles, respectively. The initial strength enhancement was primarily attributed to the optimization of the microstructure and pore structure, with a reduction in the number and width of microcracks, thereby improving mechanical performance. Additionally, within the freeze–thaw temperature range of −20 °C to 30 °C, the positive contribution of high temperatures to strength in the early stages outweighed the deteriorating effect of low temperatures, further promoting strength gain. However, after 10 freeze–thaw cycles, the positive effect of high temperatures was insufficient to offset the internal structural damage, and the propagation of microcracks led to a decrease in strength [34].

**Figure 7:**
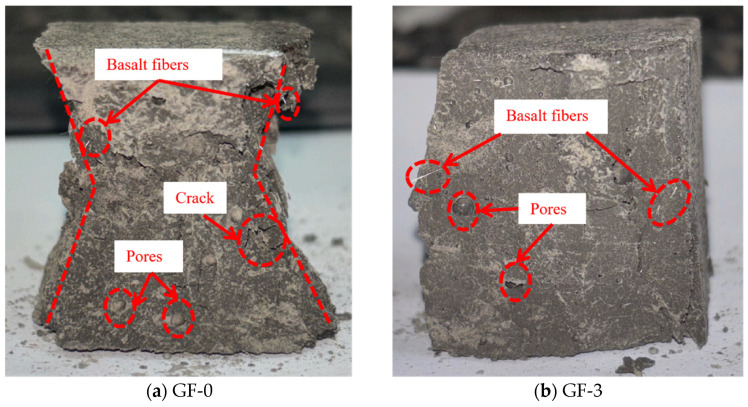

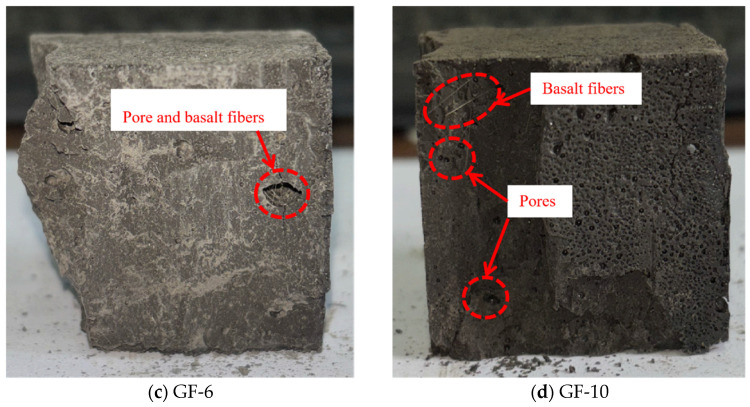
Failure modes of basalt fiber-reinforced LRS geopolymer compressive specimens after different numbers of freeze–thaw cycles.

The failure modes of basalt fiber-reinforced LRS geopolymer specimens under compression after different numbers of freeze–thaw cycles are presented in Figure 7. The control group specimens exhibited a pronounced confining effect, accompanied by considerable spalling of fragments around the edges. As the number of freeze–thaw cycles increased, the confining effect gradually diminished, and fragment spalling decreased. After 10 freeze–thaw cycles, the confining effect re-emerged, indicating that the incorporation of fibers effectively enhanced the frost resistance of the material. The fibers played a bridging role within the matrix, thereby maintaining structural integrity and stability. During uniaxial compression, as the load increased, a muffled sound was emitted from the specimens, signifying the initiation and propagation of cracks. In contrast to the unreinforced specimens, the fiber-reinforced specimens did not immediately lose their load-bearing capacity after the muffled sound; instead, they continued to sustain loading until the peak stress was reached, demonstrating that the fibers delayed the failure process even after cracking occurred. Following multiple muffled sounds, the specimens ultimately became unstable; however, no significant penetrating cracks were observed, and the internal structure remained relatively intact. Overall, although the basalt fiber-reinforced LRS geopolymer still exhibited brittle failure, its stress–strain curve displayed a “slender” shape, and the failure process demonstrated certain ductile characteristics compared to that of the unreinforced specimens.

**Figure 8:**
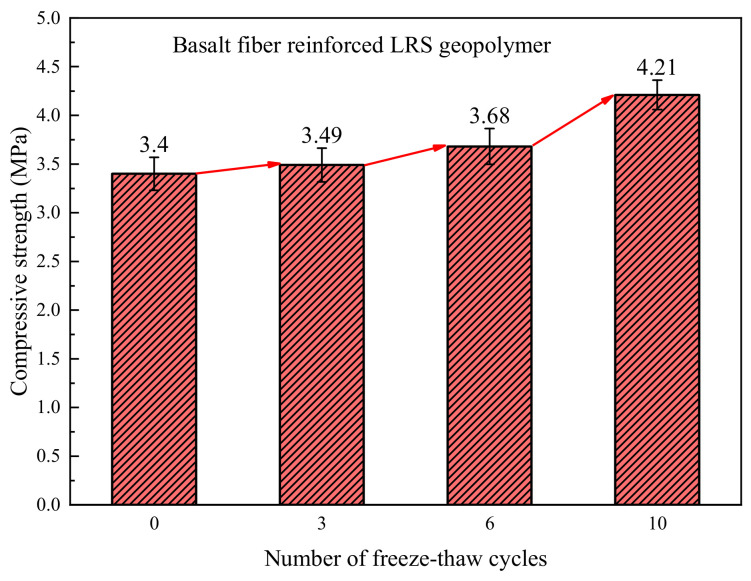
Compressive strength of basalt fiber-reinforced LRS geopolymer after different numbers of freeze–thaw cycles.

The compressive strength of basalt fiber-reinforced LRS geopolymer after different numbers of freeze–thaw cycles is shown in Figure 8. The overall strength ranges from 3.40 to 4.20 MPa, exhibiting a continuous increasing trend with the rising number of freeze–thaw cycles. Compared with the control group, after 3, 6, and 10 freeze–thaw cycles, the strength increased by 2.6%, 8.2%, and 23.5%, respectively, indicating that under the experimental conditions, freeze–thaw cycles did not weaken the compressive performance of the material but instead played a reinforcing role. Within the freeze–thaw temperature range of −20 °C to 30 °C, the positive contribution of high temperature to the strength exceeded the degradation effect caused by low temperature, further promoting the improvement of compressive performance.

#### 3.1.2. Flexural Strength and Failure Mode

**Figure 9:**
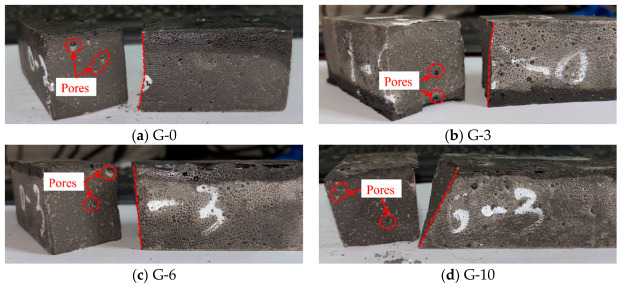
Failure modes of unreinforced LRS geopolymer flexural specimens after different numbers of freeze–thaw cycles.

The failure modes of unreinforced LRS geopolymer flexural specimens after different numbers of freeze–thaw cycles are shown in Figure 9. During the flexural tests, both the control group and the specimens subjected to freeze–thaw cycles emitted a crisp sound as the load increased, after which cracks rapidly propagated into penetrating cracks. The specimens subsequently fractured into two parts and lost their load-bearing capacity, exhibiting typical brittle failure. As the number of freeze–thaw cycles increased, the fracture process became more rapid and abrupt, with instantaneous failure occurring immediately after crack initiation. Observations of the fracture surfaces revealed that freeze–thaw cycling led to an increase in both the size and number of micropores within the geopolymer matrix, which was identified as the primary cause of the degradation in flexural performance and the reduction in ductility.

**Figure 10:**
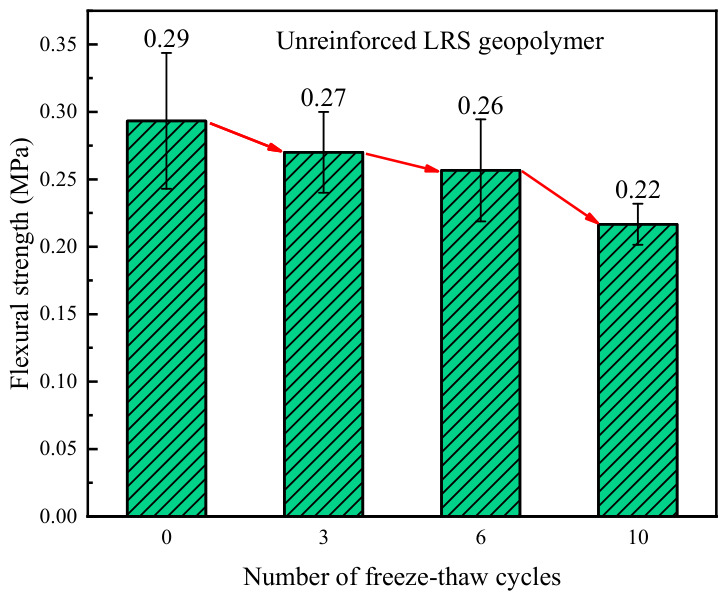
Flexural strength of unreinforced LRS geopolymer after different numbers of freeze–thaw cycles.

The flexural strength of unreinforced LRS geopolymer after different numbers of freeze–thaw cycles is presented in Figure 10. The flexural strength ranged from 0.22 to 0.29 MPa. As the number of freeze–thaw cycles increased, the flexural strength exhibited a continuous decreasing trend, with the rate of decline gradually accelerating. Compared with the control group, the flexural strength decreased by 6.9%, 10.3%, and 24.1% after 3, 6, and 10 freeze–thaw cycles, respectively, indicating that freeze–thaw action significantly degraded the flexural performance of the geopolymer. The primary reason for the strength reduction was attributed to the cumulative damage induced by repeated loading–unloading stresses resulting from pore-water expansion during freeze–thaw cycles, when the detrimental effects exceeded the material’s inherent resistance, disintegration of internal micro-elements occurred, which subsequently developed into microcracks, ultimately leading to a significant decrease in flexural strength [35,36].

**Figure 11:**
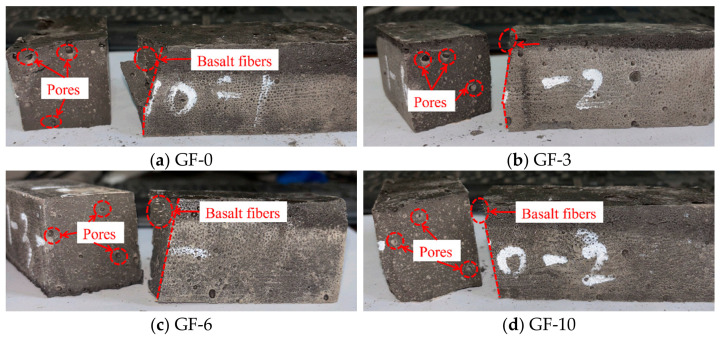
Failure modes of basalt fiber-reinforced LRS geopolymer flexural specimens after different numbers of freeze–thaw cycles.

The failure modes of basalt fiber-reinforced LRS geopolymer flexural specimens after different numbers of freeze–thaw cycles are shown in Figure 11. During the flexural tests, as the load increased, the specimens emitted a slight muffled sound, and cracks appeared. However, the cracks did not rapidly propagate into penetrating cracks. After reaching the peak strength, the specimens did not fail immediately but experienced a buffering stage during which they maintained a relatively high load-bearing capacity before eventually fracturing into two parts. Although the failure was still characterized as brittle overall, the ductility was superior to that of the unreinforced specimens. As the number of freeze–thaw cycles increased, the number of micropores on the fracture surfaces increased, though no significant enlargement of pore size was observed. Basalt fibers were clearly distinguishable on the fracture surface, indicating that the fibers effectively improved the internal structure of the geopolymer and mitigated the damage to flexural performance caused by freeze–thaw cycles.

**Figure 12:**
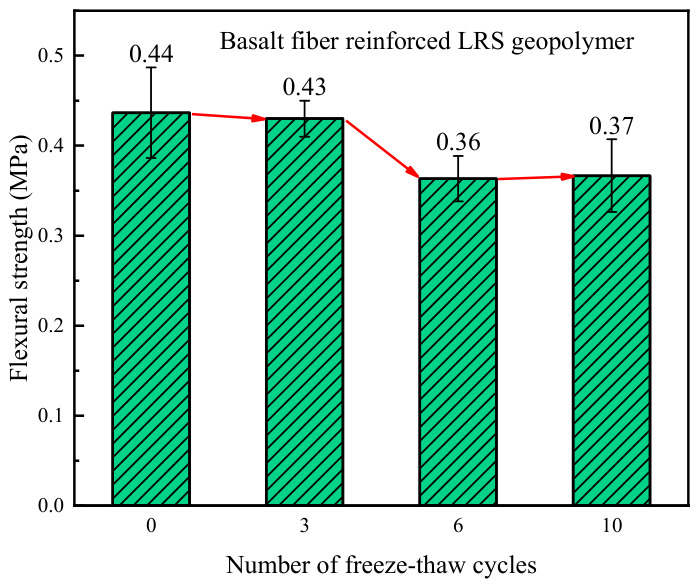
Flexural strength of basalt fiber-reinforced LRS geopolymer after different numbers of freeze–thaw cycles.

The flexural strength of basalt fiber-reinforced LRS geopolymer after different numbers of freeze–thaw cycles is shown in Figure 12. The flexural strength values were generally distributed between 0.37 and 0.44 MPa. As the number of freeze–thaw cycles increased, the flexural strength exhibited an initial decrease followed by a tendency to stabilize. Compared with the control group, the flexural strength decreased by 2.3%, 18.2%, and 15.9% after 3, 6, and 10 freeze–thaw cycles, respectively. After three freeze–thaw cycles, the strength remained nearly equivalent to that of the control group, indicating that the fibers effectively fulfilled their bridging role and maintained the flexural performance. After six cycles, the accumulation of microcracks led to partial damage to the internal structure, resulting in a more pronounced decline in strength. Following ten cycles, the strength was comparable to that observed after six cycles, suggesting that the internal structure stabilized after a certain degree of damage and that the flexural performance did not undergo further continuous deterioration.

### 3.2. Stress–Strain Behavior

#### 3.2.1. Stress–Strain Curve

**Figure 13:**
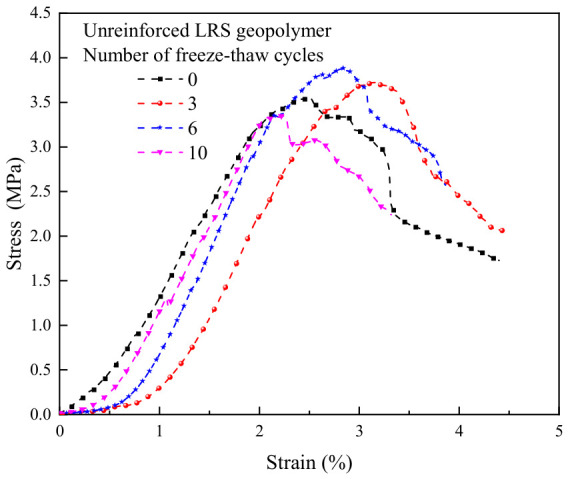
Stress–strain curve of unreinforced LRS geopolymer.

The stress–strain curves of unreinforced LRS geopolymer after different numbers of freeze–thaw cycles are presented in Figure 13. The curves exhibited a “slender” shape overall, characteristic of typical brittle failure. As the number of freeze–thaw cycles increased, the peak point first shifted to the right and then to the left, indicating that the peak strain initially increased and subsequently decreased. The peak strength also followed a trend of initial increase followed by a decrease, with the strength after the decline falling below that of the control group. This suggested that a limited number of freeze–thaw cycles could enhance strength, but further cycling led to internal damage and strength deterioration. The rapid decline in stress after reaching the peak further confirmed the brittle failure characteristics of the material. After freeze–thaw cycling, the yield point occurred earlier, reflecting enhanced brittleness and diminished ductility. Following 10 freeze–thaw cycles, the area under the curves was significantly reduced, indicating a marked decrease in the toughness of the geopolymer.

**Figure 14:**
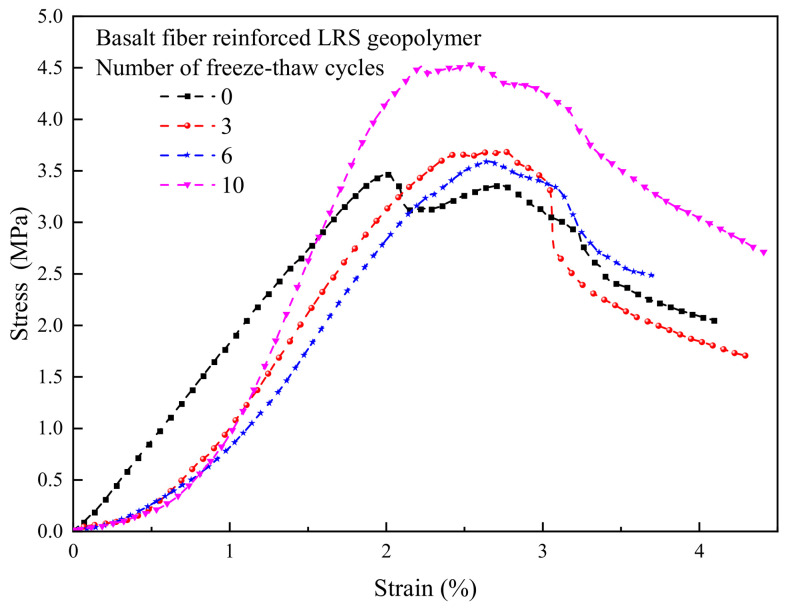
Stress–strain curve of basalt fiber reinforced LRS geopolymer.

The stress–strain curves of basalt fiber-reinforced LRS geopolymer after different numbers of freeze–thaw cycles are presented in Figure 14. The curves exhibited a “slender” shape overall, still reflecting brittle failure characteristics. As the number of freeze–thaw cycles increased, the peak point initially shifted to the right and then to the left; however, the peak strain for all freeze–thaw groups remained higher than that of the control group. The peak strength was also higher than that of the control group, indicating that freeze–thaw cycling enhanced the compressive performance of the fiber-reinforced geopolymer. After reaching the peak strength, a distinct “plateau region” appeared in the curves, where the stress decreased relatively slowly. This behavior reflected the bridging effect of the fibers during crack propagation, which delayed the failure process. As loading continued, the internal structure ultimately failed, leading to a more rapid stress decline and convergence of the curves. Following freeze–thaw cycling, the area under the curves increased, indicating an improvement in toughness.

#### 3.2.2. Stress–Strain Model

**Figure 15:**
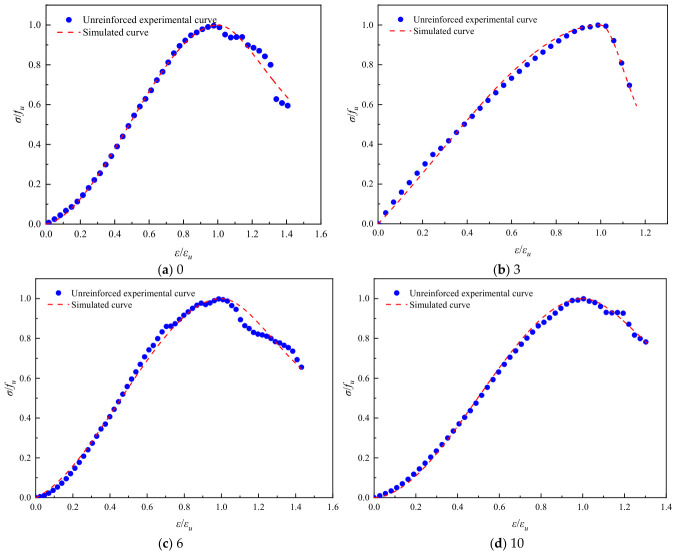
Comparison of fitting curves based on the Guo Zhenhai model for unreinforced LRS geopolymer under different numbers of freeze–thaw cycles.

**Figure 16:**
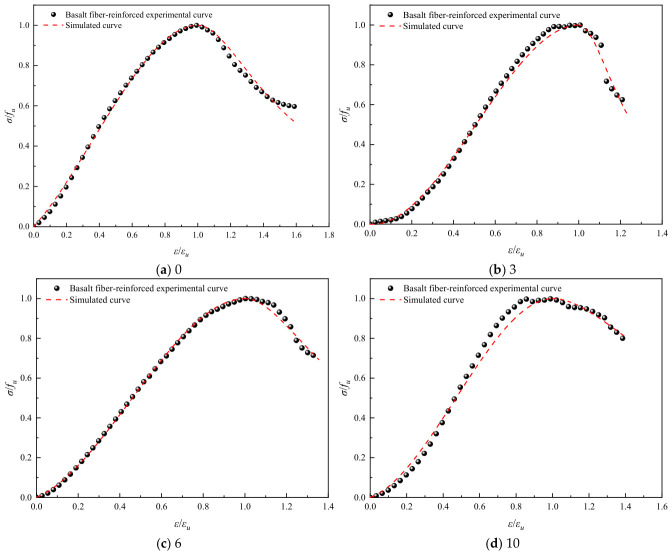
Comparison of fitting curves based on the Guo Zhenhai model for basalt fiber-reinforced LRS geopolymer under different numbers of freeze–thaw cycles.

**Figure 17:**
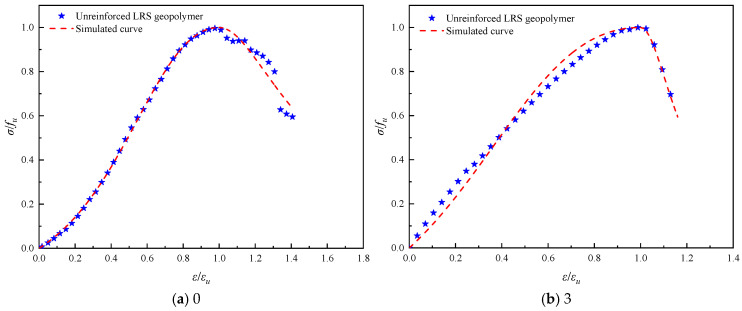

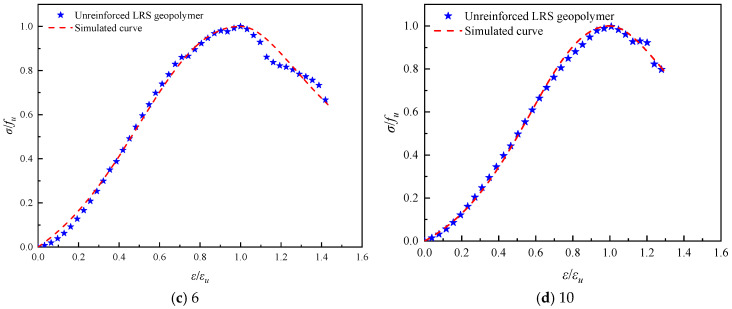
Comparison of fitting curves based on the Saenz L P model for unreinforced LRS geopolymer under different numbers of freeze–thaw cycles.

**Figure 18:**
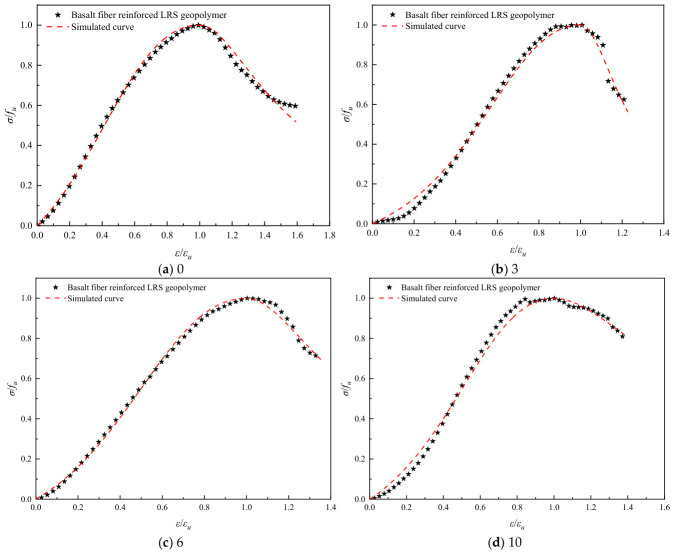
Comparison of fitting curves based on the Saenz L P model for basalt fiber-reinforced LRS geopolymer under different numbers of freeze–thaw cycles.

**Figure 19:**
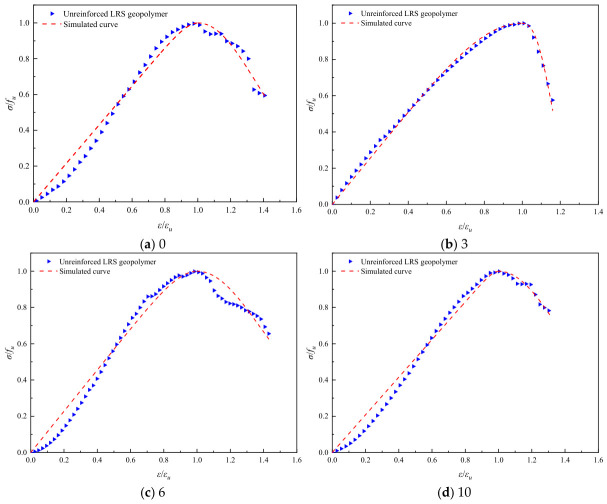
Comparison of fitting curves based on the Carrerira D J model for unreinforced LRS geopolymer under different numbers of freeze–thaw cycles.

**Figure 20:**
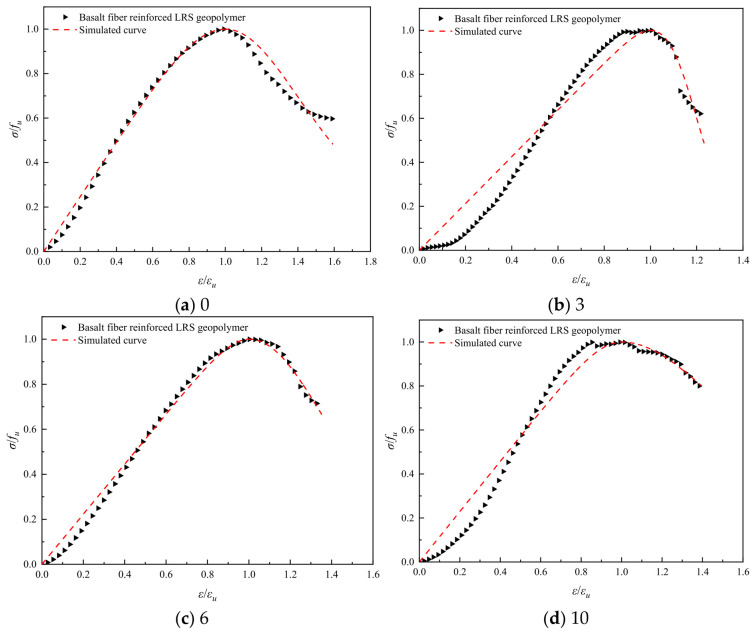
Comparison of fitting curves based on the Carrerira D J model for basalt fiber-reinforced LRS geopolymer under different numbers of freeze–thaw cycles.

Referring to common constitutive models for ordinary concrete, such as the Guo Zhenhai model [37], the Saenz L P model [38], and the Carrerira D J model [39], the model equations are presented in Table 4. After normalizing the stress–strain curves, the aforementioned models were employed to fit both the ascending and descending branches of the geopolymer stress–strain curves after different numbers of freeze–thaw cycles. The fitting results are summarized in Table 5. Comparisons between the normalized experimental full curves and the theoretical fitted curves for each model are shown in Figure 15, Figure 16, Figure 17, Figure 18, Figure 19 and Figure 20.

**Table 4:** Full curve model of concrete stress–strain.

Model	Equation	Coefficient	
Saenz L.P. model [38]	$y=\frac{mx}{1+x(m-2)+x^{2}}$	*m*	(3)
Carreira D.J. model [39]	$y=\frac{mx}{m-1+x^{m}}$	*m*	(4)
Guo Zhenhai model [37]	$y=mx+(3-2m)x^{2}+(m-2)x^{3},0\leq x<1$ $y=\frac{x}{m{\left(x-1\right)}^{2}+x},x\geq 1$	*m*	(5)

Note: *y = σ/f_u_*; *σ* is stress; *f_u_* is peak stress; *x = ε/ε_u_*; *ε* is the strain; *ε_u_* is the peak strain; *m* is the shape factor of the model.

**Table 5:** Fitting values of the shape coefficient for the LRS geopolymer stress–strain curve model.

Model	Order of Curve	Number of Freeze–Thaw Cycles	0		3		6		10	
		Whether Fiber is Added	No	Yes	No	Yes	No	Yes	No	Yes
Saenz L.P. model [38]	Rising section	*m*	0.523	0.830	0.955	0.464	0.629	0.614	0.470	0.604
		*R* ^2^	0.999	0.997	0.984	0.994	0.997	0.998	0.997	0.991
	Falling section	*m*	0.204	0.237	0.032	0.054	0.237	0.210	3.980	0.477
		*R* ^2^	0.873	0.947	0.987	0.937	0.768	0.955	0.930	0.938
Carreira D.J. model [39]	Rising section	*m*	13.784	5.319	4.611	16.565	8.269	9.902	27.233	7.983
		*R* ^2^	0.980	0.994	0.996	0.948	0.977	0.989	0.979	0.961
	Falling section	*m*	6.869	5.681	22.654	15.308	6.062	6.963	6.455	4.310
		*R* ^2^	0.913	0.844	0.973	0.854	0.627	0.974	0.928	0.943
Guo Zhenhai model [37]	Rising section	*m*	0.182	0.912	1.174	−0.091	0.434	0.444	0.044	0.333
		*R* ^2^	0.999	0.998	0.995	0.997	0.997	0.999	0.997	0.989
	Falling section	*m*	4.903	4.218	30.831	18.514	4.223	4.765	3.980	2.096
		*R* ^2^	0.873	0.947	0.987	0.938	0.768	0.955	0.931	0.939

Among the three concrete constitutive models mentioned above, the shape coefficient *m* is the core dimensionless parameter that determines the geometric characteristics of the uniaxial compressive stress–strain curve. Its physical significance lies in controlling the evolution rate and morphology of the curve from the linear elastic stage through the peak stress to the post-peak softening stage. In the ascending branch (*x* < 1), *m* mainly influences the initial tangent slope of the curve and the sharpness of the peak. Taking the Guo Zhenhai model as an example, where a cubic polynomial is used for the ascending branch, a larger *m* results in a steeper initial slope, indicating more pronounced linear elastic behavior and an abrupt peak failure. Conversely, a smaller *m* leads to a gentler ascending curve and a more “flattened” region near the peak, reflecting an enhanced plastic deformation capacity of the material before reaching the ultimate strength. In the descending branch (*x* ≥ 1), a larger *m* means a more significant contribution from the quadratic term in the denominator, causing the post-peak stress to decay more rapidly with increasing strain. The curve exhibits a “steep drop” characteristic, representing sudden brittle failure. In contrast, a smaller *m* results in a relatively gentler post-peak curve, indicating certain residual strength and ductility. The shape coefficient *m* in the Saenz and Carreira models serves a similar function. Therefore, the shape coefficient *m* can be regarded as a mathematical mapping of the internal structural state of the material to its macroscopic mechanical response.

From the fitting results presented in Table 5, it can be observed that for the ascending branches of the curves, the correlation coefficients (*R*^2^) ranked as follows: Guo Zhenhai model > Saenz L P model > Carrerira D J model. For the descending branches, the *R*^2^ values followed the same order: Guo Zhenhai model > Saenz L P model > Carrerira D J model. A comparative analysis of the correlation coefficients of the three models indicated that both the ascending and descending branches fitted by the Guo Zhenhai model exhibited better agreement with the stress–strain curves of the alkali-activated basalt fiber-reinforced LRS geopolymer. Furthermore, a comparison of the fitted curves from each model, as shown in Figure 15, Figure 16, Figure 17, Figure 18, Figure 19 and Figure 20, revealed that throughout the freeze–thaw cycle tests, both the ascending and descending branches of the Guo Zhenhai model demonstrated closer alignment with the experimental stress–strain curves of the geopolymer.

Based on the comprehensive experimental results and incorporating statistical patterns derived from the data, the reference values for the peak strain of basalt fiber-reinforced LRS geopolymer are summarized in Table 6.

The Guo Zhenhai model provides a better fit because it adopts a piecewise function formulation—the ascending branch is a polynomial that describes the progressive damage characteristics from linear elasticity to plasticity, while the descending branch is a rational fraction that better captures the physical reality of the sharp post-peak stress drop of LRS geopolymers as a brittle material after freeze–thaw cycles. In contrast, the continuous function form of the Saenz model cannot accurately capture the post-peak stiffness degradation, and the Carreira model lacks flexibility in fitting the descending branch. Furthermore, the influence of fiber reinforcement and freeze–thaw damage on the shape parameters is as follows: For the unreinforced group, the shape parameters exhibit fluctuating trends, reflecting the competition between microcrack propagation and localized self-healing during freeze–thaw cycles. For the fiber-reinforced group, the shape parameters show a monotonic trend, indicating that the fiber bridging effect suppresses disordered crack propagation and leads to more stable damage evolution.

#### 3.2.3. Elastic Modulus

**Figure 21:**
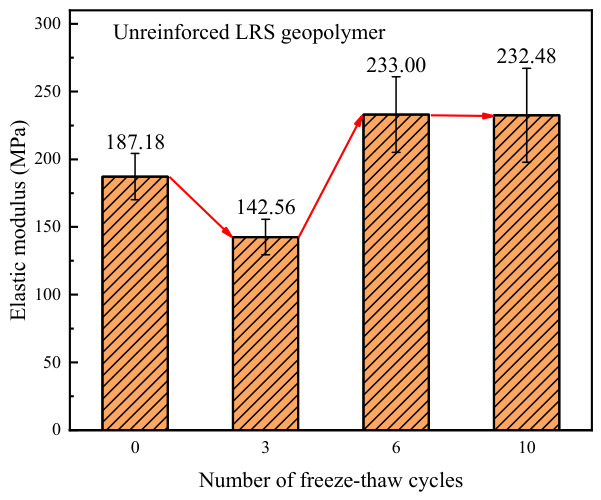
Elastic modulus of the unreinforced LRS geopolymer.

The elastic modulus of unreinforced LRS geopolymer is shown in Figure 21, with values ranging from 142.56 to 233.00 MPa. As the number of freeze–thaw cycles increased, the elastic modulus exhibited an initial decrease, followed by an increase and subsequent stabilization. Compared with the control group, the elastic modulus of the specimens changed by −23.8%, +24.5%, and +24.2% after 3, 6, and 10 freeze–thaw cycles, respectively. This indicates that under the effect of multiple freeze–thaw cycles, the elastic modulus of unreinforced LRS geopolymer improved and eventually stabilized. However, at the same time, the peak strain decreased and ductility diminished, suggesting that the material failed at a faster rate upon reaching failure.

**Figure 22:**
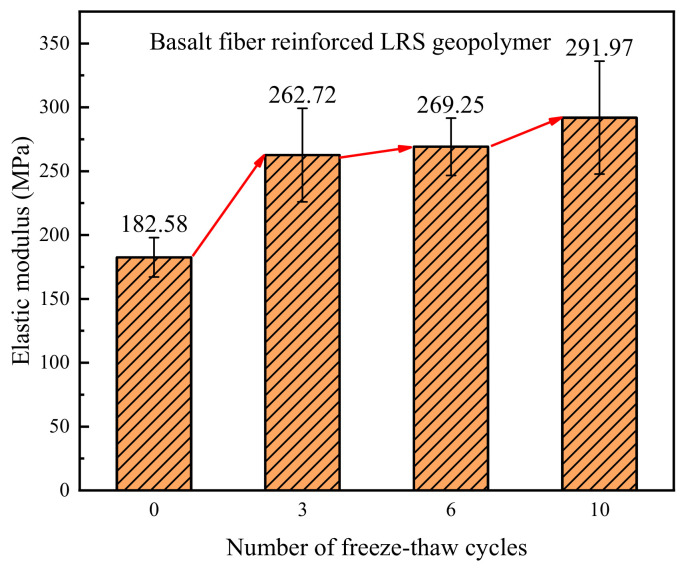
Elastic modulus of the basalt fiber-reinforced LRS geopolymer.

The elastic modulus of basalt fiber-reinforced LRS geopolymer is presented in Figure 22, with values ranging from 182.58 to 291.97 MPa. As the number of freeze–thaw cycles increased, the elastic modulus exhibited a gradually increasing trend. Compared with the control group, the elastic modulus of the specimens increased by 43.9%, 47.5%, and 59.9% after 3, 6, and 10 freeze–thaw cycles, respectively. Under the alternating influence of high and low temperatures, the unreinforced LRS geopolymer experienced performance degradation, whereas the basalt fiber-reinforced LRS geopolymer effectively mitigated the performance damage caused by freeze–thaw cycles, maintaining the stability of the internal structure of the material and thereby preventing the attenuation of the elastic modulus.

#### 3.2.4. Peak Strain

**Figure 23:**
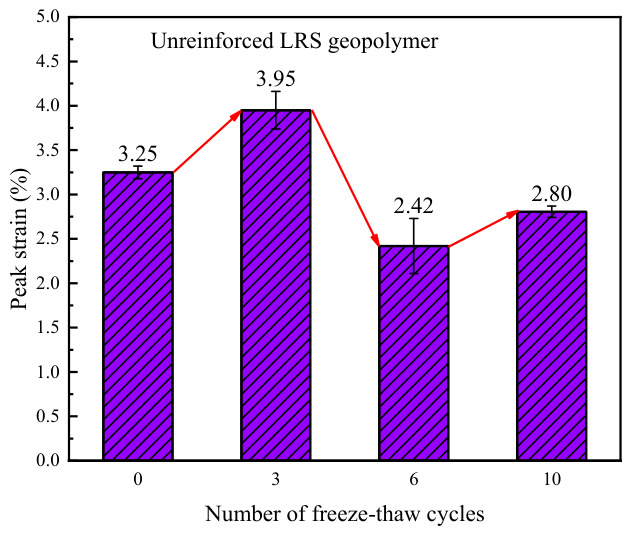
Peak strain of the unreinforced LRS geopolymer.

The peak strain of unreinforced LRS geopolymer is shown in Figure 23. The data indicate that the peak strain ranged from 2.42% to 3.95%, exhibiting a fluctuating trend of “increase–decrease–increase” as the number of freeze–thaw cycles increased, which was inversely correlated with the evolution pattern of the elastic modulus. Compared with the control group, the peak strain changed by +21.5%, −25.5%, and −13.8% after 3, 6, and 10 freeze–thaw cycles, respectively. The decrease in peak strain during the later stages of freeze–thaw cycling was primarily attributed to two factors. On the one hand, the increase in elastic modulus accelerated the linear elastic stage, causing the material to enter the plastic stage earlier and consequently reducing the peak strain. On the other hand, changes in the state of pore water during freeze–thaw cycles led to an increase in both the number and size of internal pores, which weakened the plastic deformation capacity of the material.

**Figure 24:**
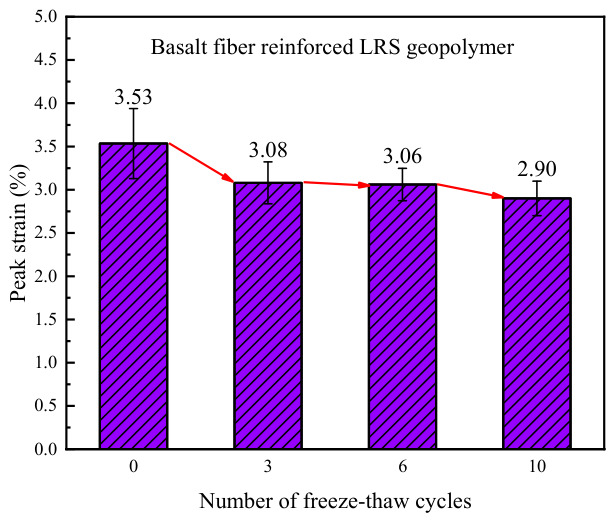
Peak strain of the basalt fiber-reinforced LRS geopolymer.

The peak strain of the basalt fiber-reinforced LRS geopolymer is presented in Figure 24, with values ranging from 2.90% to 3.53%. As the number of freeze–thaw cycles increased, the peak strain exhibited a gradually decreasing trend. Compared with the control group, the peak strain of the specimens decreased by 5.2%, 5.8%, and 10.7% after 3, 6, and 10 freeze–thaw cycles, respectively. This phenomenon was primarily attributed to the following reasons: as the number of freeze–thaw cycles increased, the elastic modulus of the material continuously increased, accelerating the rate of strength development during the linear elastic stage and thereby causing the peak strength to be reached earlier. Concurrently, the plastic deformation capacity of the geopolymer diminished after freeze–thaw cycling, which, together with the reduction in peak strain, contributed to the reduction in peak strain.

The 3.53% peak strain in Figure 24 is the compressive failure strain of the composite, not the fracture strain of basalt fibers. In short-fiber-reinforced brittle composites, fiber pull-out and interfacial debonding can lead to composite failure strain exceeding that of the fibers. The fiber properties in Table 2 are from monofilament tests; the actual fiber bundles (rovings) have a lower fracture strain of 2.5–3.0% due to asynchronous filament fracture. For specimens without freeze–thaw cycles, weak interfacial bonding allowed significant fiber pull-out, resulting in 3.53% strain. With increasing freeze–thaw cycles, interfacial bonding improved, fiber pull-out was restricted, and the ultimate strain decreased to below 3%. Thus, the 3.53% deformation is a reasonable phenomenon governed by fiber-matrix interfacial behavior.

### 3.3. Discussions on Effects of Basalt Fiber Reinforcement

**Figure 25:**
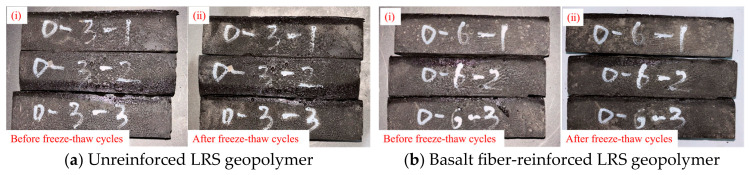
Freeze–thaw cycle 3 times.

**Figure 26:**
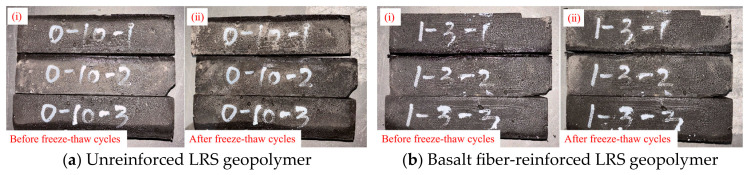
Freeze–thaw cycle 6 times.

**Figure 27:**
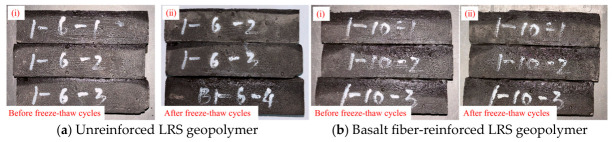
Freeze–thaw cycle 10 times.

The comparison of apparent characteristics of geopolymers before and after freeze–thaw cycles is shown in Figure 25, Figure 26 and Figure 27. Compared with the undisturbed geopolymer not subjected to freeze–thaw cycles, the surface color of the geopolymer after freeze–thaw cycling appeared darker. After multiple freeze–thaw cycles, the unreinforced LRS geopolymer specimens exhibited slight shape deformation. In contrast, the basalt fiber-reinforced LRS geopolymer showed negligible changes in appearance after the same number of cycles.

**Figure 28:**
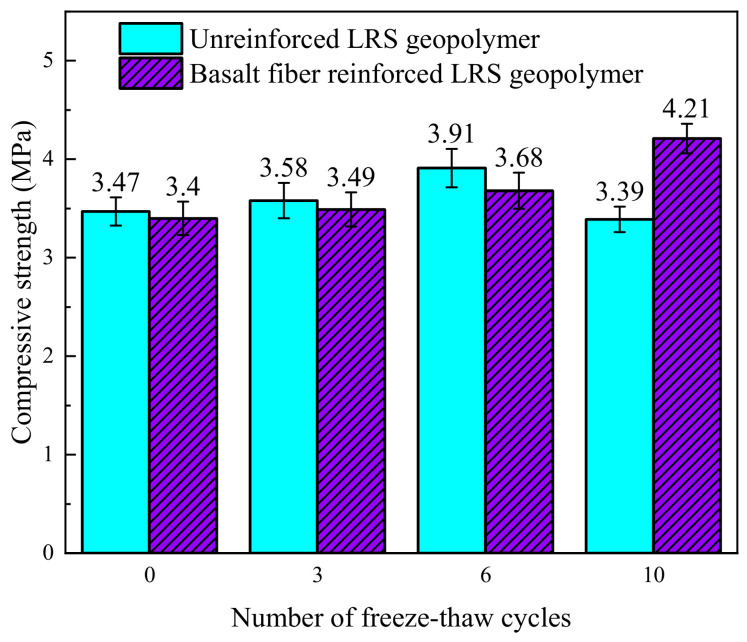
Comparison of the compressive strength of LRS geopolymers after freeze–thaw cycles.

A comparison of the compressive strength of LRS geopolymers after freeze–thaw cycles is presented in Figure 28. During the 0 to 6 cycles, the compressive strength of the LRS geopolymer without fiber addition was higher than that of the basalt fiber-reinforced group. This is because the incorporation of fibers reduced the fluidity of the paste and introduced some pores, which was detrimental to the improvement of compressive strength [40]. However, after 10 freeze–thaw cycles, the strength of the unreinforced group decreased and fell below that of the control group. In contrast, the strength of the fiber-reinforced group continued to increase, showing a 23% improvement compared to the unreinforced group. These observations indicate that under repeated freeze–thaw cycles, the internal structure of the unreinforced group underwent degradation. In contrast, the structure of the fiber-reinforced group remained relatively intact, enabling its compressive performance to continue to improve. Regarding the phenomenon of continuous strength growth in the fiber-reinforced group after multiple cycles, it is hypothesized that this may be related to the warming phase during the cycling process.

A comparison of the failure modes of LRS geopolymer compressive specimens after freeze–thaw cycles is shown in Figure 5 and Figure 7. The control specimens exhibited a pronounced confining effect; however, as the number of freeze–thaw cycles increased, this effect gradually diminished and eventually disappeared. During uniaxial compression, the unreinforced LRS geopolymer specimens emitted a sharp cracking sound, accompanied by considerable spalling of fragments around the edges. They rapidly lost their load-bearing capacity immediately after the sound occurred. In contrast, the basalt fiber-reinforced specimens produced a more muffled sound. Following this muffled sound, the specimens had not yet reached their peak stress and were able to continue sustaining the load; failure ultimately occurred only after multiple muffled sounds. After freeze–thaw cycling, distinct penetrating cracks were observed in the unreinforced specimens, leading to failure. In contrast, no obvious penetrating cracks were detected in the fiber-reinforced specimens, indicating that the bridging effect of the fibers contributed to a more compact internal structure. It should be noted that this study did not conduct microstructural characterization (e.g., SEM). However, reasonable explanations for the above macroscopic phenomena can be provided based on microstructural evidence from the existing literature. Zhang et al. [34] confirmed the presence of fiber pull-out traces and surface abrasion after freeze–thaw cycles, verifying the energy dissipation effect of fiber–matrix friction. Pang et al. [41] presented SEM images showing that fibers effectively bridge microcracks and inhibit their propagation. Debbarma et al. [40] demonstrated that fibers can form a three-dimensional network structure in LRS geopolymers, bridging pores and microcracks. Based on these findings, it can be inferred that the observed improvements in freeze–thaw durability of the fiber-reinforced specimens in this study—such as the absence of penetrating cracks, enhanced ductility, and stabilization of flexural strength—can be attributed to the bridging effect of the basalt fibers. Both types of specimens exhibited audible sounds and fragment spalling during the failure process and lost their load-bearing capacity relatively quickly after reaching peak strength; therefore, both were characterized as brittle failures.

**Figure 29:**
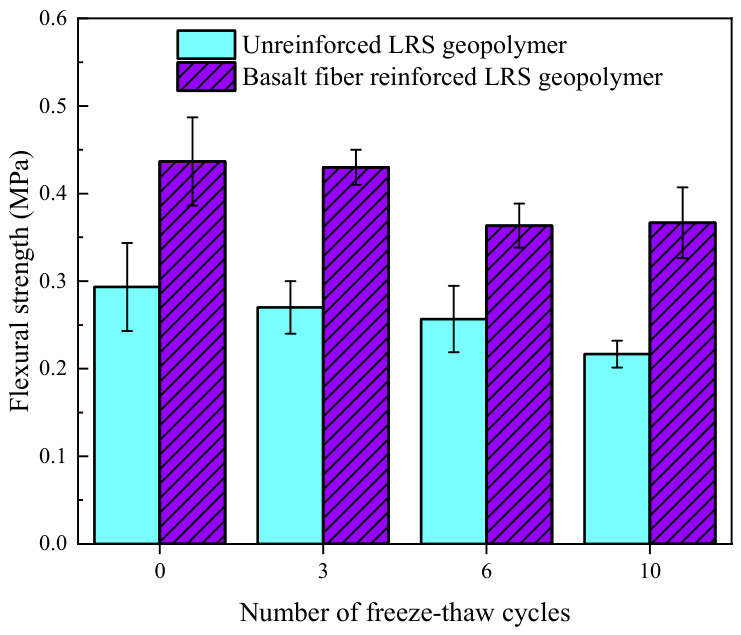
Comparison of flexural strength of LRS geopolymers after freeze–thaw cycles.

A comparison of the flexural strength of LRS geopolymers after freeze–thaw cycles is shown in Figure 29. Overall, the flexural strength of the basalt fiber-reinforced specimens was consistently higher than that of the unreinforced specimens. As the number of freeze–thaw cycles increased, the flexural strength of the unreinforced specimens exhibited a continuous decline, whereas that of the fiber-reinforced specimens remained constant. However, decreasing gradually tended to stabilize. This indicated that the incorporation of fibers significantly enhanced the flexural performance of the geopolymer. Compared with compressive performance, flexural performance was more sensitive to freeze–thaw cycles.

A comparison of the failure modes of LRS geopolymer flexural specimens after freeze–thaw cycles is presented in Figure 9 and Figure 11. During flexural loading, the unreinforced specimens emitted a sharp cracking sound, after which cracks rapidly propagated into penetrating cracks, leading to the specimens fracturing into two parts and losing their load-bearing capacity, which was characteristic of typical brittle failure. In contrast, the basalt fiber-reinforced specimens emitted multiple slight muffled sounds accompanied by crack initiation, but the cracks did not rapidly propagate into penetrating cracks. After reaching the ultimate strength, the specimens did not fail immediately; instead, they experienced a buffering stage during which they maintained a relatively high load-bearing capacity before eventual failure occurred. Although the failure remained brittle in nature, the ductility of the fiber-reinforced specimens was noticeably superior to that of the unreinforced ones. The incorporation of basalt fibers effectively prevented the rapid propagation of cracks, partially offset the tensile stress in the tension zone, and improved the internal structure of the geopolymer, enabling it to remain relatively dense after freeze–thaw cycling. This, in turn, reduced performance degradation and enhanced both ductility and frost resistance.

A comparison of the stress–strain curves of LRS geopolymers after freeze–thaw cycles is shown in Figure 13 and Figure 14. All curves exhibited a slender shape overall. As the number of freeze–thaw cycles increased, the peak point of the unreinforced LRS geopolymer first shifted to the right and then to the left, indicating that the peak strain initially increased and then decreased. In contrast, the peak strength first increased and subsequently decreased. For the basalt fiber-reinforced LRS geopolymer, the peak point first gradually shifted to the right and then slightly to the left, with an overall increase in peak strain and a continuous increasing trend in peak strength. The incorporation of fibers significantly extended the plastic region of the stress–strain curves and increased plastic deformation, enabling the specimens to maintain a relatively high load-bearing capacity after reaching ultimate strength and to delay fracture. Compared with the unreinforced group, the fiber-reinforced group exhibited a longer yield stage and less influence on the plastic region after freeze–thaw cycling, indicating that the fibers effectively inhibited rapid crack propagation and enhanced both the ductility and frost resistance of the geopolymer. Furthermore, the area under the stress–strain curves for the fiber-reinforced group was markedly larger than that for the unreinforced group, demonstrating an improvement in toughness as well.

**Figure 30:**
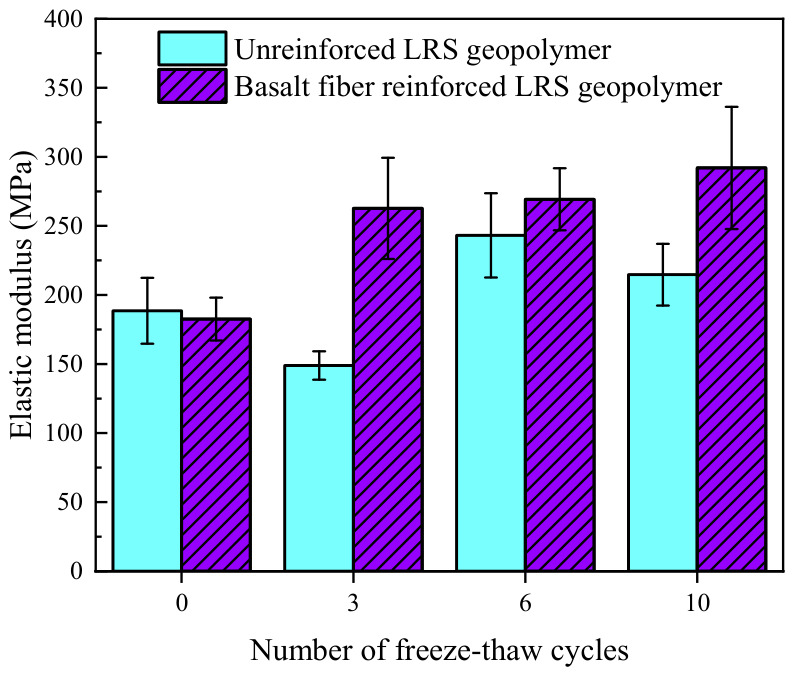
Comparison of elastic modulus of LRS geopolymers after freeze–thaw cycles.

A comparison of the elastic modulus of LRS geopolymers after freeze–thaw cycles is presented in Figure 30. Before freeze–thaw cycling, the elastic modulus of the unreinforced LRS geopolymer was slightly higher than that of the basalt fiber-reinforced group. After freeze–thaw cycling, the elastic modulus of the fiber-reinforced group became significantly higher than that of the unreinforced group. As the number of freeze–thaw cycles increased, the elastic modulus of the unreinforced group exhibited a fluctuating trend of “decrease–increase–decrease”. Although the growth rate gradually slowed down, the fiber-reinforced group continued to show a sustained increasing trend.

**Figure 31:**
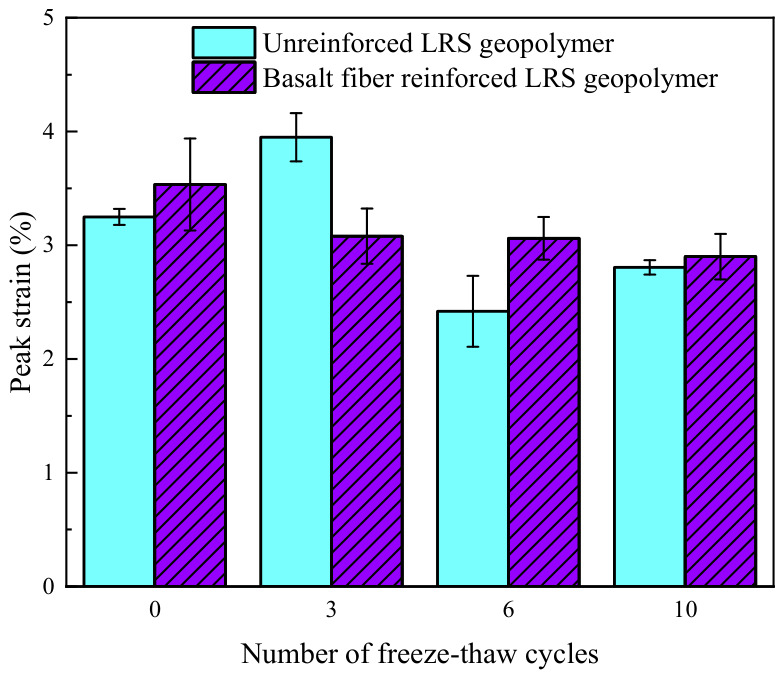
Comparison of the peak strain of LRS geopolymers after freeze–thaw cycles.

A comparison of the peak strain of LRS geopolymers after freeze–thaw cycles is presented in Figure 31. The peak strain of the unreinforced LRS geopolymer was significantly influenced by alternating high and low temperatures, exhibiting pronounced instability. In contrast, although the peak strain of the basalt fiber-reinforced LRS geopolymer gradually decreased with increasing numbers of freeze–thaw cycles, the rate of decrease progressively diminished and eventually stabilized within a predictable range. A comparison of the trends in elastic modulus and peak strain revealed that the two parameters generally exhibited an inverse relationship, indicating that the elastic modulus had a significant influence on the peak strain. After freeze–thaw cycling, the plastic region in the stress–strain curves of the geopolymer was adversely affected, manifesting as a narrowing of the plastic region and a reduction in plastic deformation. The incorporation of basalt fibers effectively mitigated this deterioration trend and alleviated the extent of reduction in plastic deformation, thereby resulting in a more gradual decline in the peak strain of the basalt fiber-reinforced LRS geopolymer.

To quantify the degree of damage to the flexural strength of LRS geopolymer induced by freeze–thaw cycles, the flexural strength damage variable was defined using the formula shown in Equation (6):

(6) $$D=(1-\frac{f_{T}}{f_{0}})\times 100\%$$

where

*D* is the reduction (loss) in flexural strength of the LRS geopolymer after freeze–thaw cycles; *f_T_* is the flexural strength of the LRS geopolymer after freeze–thaw cycles, in megapascals (MPa); *f*_0_ is the flexural strength of the LRS geopolymer before freeze–thaw cycles, in megapascals (MPa).

**Figure 32:**
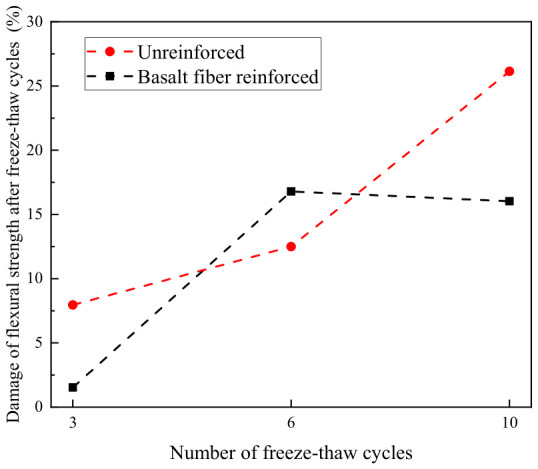
Flexural strength damage of LRS geopolymers after freeze–thaw cycles.

The damage to the flexural strength of geopolymers after freeze–thaw cycles is shown in Figure 32. For the unreinforced LRS geopolymer, the flexural strength damage after 3, 6, and 10 freeze–thaw cycles was 8.0%, 12.5%, and 26.1%, respectively, exhibiting a progressively worsening trend with an accelerating damage rate. For the basalt fiber-reinforced LRS geopolymer, the corresponding damage values were 1.5%, 16.8%, and 16.0%, characterized by a sharp increase in the early stage followed by stabilization in the later stage. After three freeze–thaw cycles, the damage in the unreinforced group had already reached 5.3 times that of the fiber-reinforced group, indicating that significant damage occurred in the unreinforced geopolymer during the early stage of freeze–thaw cycling, while the fiber-reinforced group experienced less damage with flexural strength remaining close to the reference value. This demonstrated that fiber incorporation effectively mitigated early-stage damage. After ten freeze–thaw cycles, the damage in the unreinforced group had intensified markedly, reaching 1.6 times that of the fiber-reinforced group. At this stage, the damage in the unreinforced group continued to increase, whereas that in the fiber-reinforced group had stabilized. These results indicated that after multiple freeze–thaw cycles, the incorporation of an appropriate amount of basalt fibers significantly inhibited damage to flexural strength, enabling the geopolymer to maintain satisfactory flexural performance.

To quantitatively evaluate the reinforcing effect of basalt fibers on the mechanical properties of LRS geopolymer after freeze–thaw cycles, the compressive strength gain ratio *β*_C_ and the flexural strength gain ratio *β*_F_ were introduced. The expressions are shown in Equations (7) and (8), respectively:

(7) $$\beta_{C}=\frac{f_{C}}{f_{Cg}}$$

where *β_C_* is the compressive strength gain ratio of the basalt fiber-reinforced LRS geopolymer after freeze–thaw cycles; *f_C_* is the compressive strength of the basalt fiber-reinforced LRS geopolymer after freeze–thaw cycles, in megapascals (MPa); *f_Cg_* is the compressive strength of the unreinforced LRS geopolymer after freeze–thaw cycles, in megapascals (MPa).

(8) $$\beta_{F}=\frac{f_{F}}{f_{Fg}}$$

where *β_F_* is the flexural strength gain ratio of the basalt fiber-reinforced LRS geopolymer after freeze–thaw cycles; *f_F_* is the flexural strength of the basalt fiber-reinforced LRS geopolymer after freeze–thaw cycles, in megapascals (MPa); *f_Fg_* is the flexural strength of the unreinforced LRS geopolymer after freeze–thaw cycles, in megapascals (MPa).

**Figure 33:**
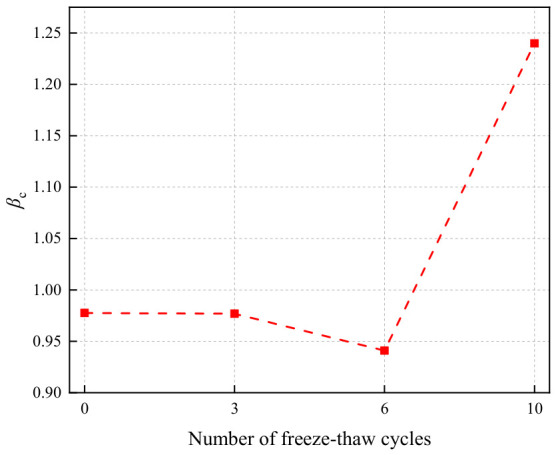
Compressive strength gain ratio of LRS geopolymers after freeze–thaw cycles.

The compressive strength gain ratio of geopolymers after freeze–thaw cycles is shown in Figure 33. As the number of freeze–thaw cycles increased, the compressive strength gain ratio exhibited a trend of initially decreasing and then sharply increasing. After 0, 3, 6, and 10 freeze–thaw cycles, the compressive strength gain ratios were 0.98, 0.98, 0.94, and 1.92, respectively, indicating that the reinforcing effect of basalt fibers on the compressive strength of the geopolymer became significantly more pronounced after multiple freeze–thaw cycles. The primary reason for this phenomenon was that after multiple freeze–thaw cycles, the internal structure of the unreinforced LRS geopolymer was substantially affected by temperature variations, exceeding the material’s inherent frost resistance capacity, leading to severe internal structural deterioration and a sharp reduction in compressive strength. In contrast, the incorporation of basalt fibers effectively improved the microstructure of the geopolymer, thereby significantly enhancing the compressive strength gain ratio.

**Figure 34:**
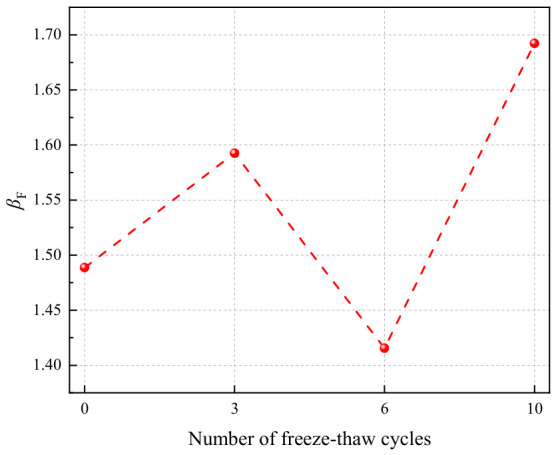
Flexural strength gain ratio of LRS geopolymers after freeze–thaw cycles.

The flexural strength gain ratio of geopolymers after freeze–thaw cycles is presented in Figure 34. As the number of freeze–thaw cycles increased, the flexural strength gain ratio exhibited a fluctuating trend of “increase–decrease–increase”. After 0, 3, 6, and 10 freeze–thaw cycles, the flexural strength gain ratios were 1.49, 1.59, 1.42, and 1.69, respectively, all exceeding 1. This indicated that the incorporation of basalt fibers effectively enhanced the flexural strength of the geopolymer at every stage of freeze–thaw cycling. Notably, when the number of freeze–thaw cycles reached 10, the flexural strength gain ratio increased significantly, demonstrating that the reinforcing effect of basalt fibers on flexural strength became more pronounced after multiple freeze–thaw cycles. The underlying reason for this observation was that the basalt fiber-reinforced LRS geopolymer possessed superior frost resistance, sustaining less damage to its mechanical properties after freeze–thaw cycling, which further highlighted its advantage in flexural strength.

## 4. Conclusions

This study investigated the mechanical properties and durability of unreinforced and basalt fiber-reinforced LRS geopolymers after freeze–thaw cycles, and established constitutive models under different numbers of freeze–thaw cycles. By comparing compressive strength, flexural strength, failure modes, stress–strain curves, elastic modulus, ultimate strain, flexural strength damage, and strength gain ratios, the reinforcing mechanism of basalt fibers on the frost resistance of the geopolymer was revealed. The core novelty of this study lies in extending the research on basalt fiber-reinforced LRS geopolymers into the field of freeze–thaw durability, and establishing for the first time a freeze–thaw damage constitutive model, thereby providing a theoretical basis for the design and service life prediction of in situ lunar construction materials. The main conclusions are as follows:

(1) Basalt fiber reinforcement consistently improved the mechanical performance of LRS geopolymers under freeze–thaw cycles. After multiple cycles, both compressive and flexural strengths of the fiber-reinforced group were higher than those of the unreinforced group, with a more pronounced gain in flexural strength. This suggests that fibers are more effective in resisting tensile stresses induced by freeze–thaw damage.
(2) Both types of geopolymers exhibited typical brittle failure characterized by “slender” stress–strain curves. However, the fiber-reinforced group showed improved post-peak ductility and fracture toughness. The uniformly distributed fibers with random orientation helped bridge microcracks, inhibit pore expansion, and enhance interfacial adhesion, thereby improving frost resistance.
(3) Basalt fibers significantly mitigated flexural strength degradation. While damage in the unreinforced group increased progressively with cycle number, the fiber-reinforced group exhibited limited damage that stabilized after multiple cycles, demonstrating the protective effect of fibers against cumulative freeze–thaw damage.
(4) After multiple cycles, the flexural strength gain ratio of the fiber-reinforced geopolymer remained consistently above 1 and peaked after 10 cycles. This indicates that the reinforcing effect of fibers on strength and ductility increases with cycle number in the early to middle stages. Furthermore, beyond a certain number of cycles, fibers also began to enhance compressive strength, suggesting a delayed but positive effect on compressive performance.
(5) Constitutive models for both types of geopolymers under different numbers of cycles were established based on the Saenz L P model, the Carrerira D J model, and the Guo Zhenhai model. Comparative analysis showed that the Guo Zhenhai model best matched the experimental stress–strain curves in both the ascending and descending branches, providing a reliable basis for future modeling of similar materials under freeze–thaw conditions.

### Limitations and Future Perspectives

It should be noted that this study has certain limitations. First, the freeze–thaw tests were conducted on water-saturated specimens under terrestrial atmospheric conditions, which did not fully replicate the vacuum, anhydrous, and low-gravity environment of the Moon. Nevertheless, the classical freeze–thaw mechanism can still provide valuable reference for understanding material behavior under extreme environments. Second, microstructural characterization (e.g., scanning electron microscopy, mercury intrusion porosimetry) was not performed in this study. Therefore, the mechanistic interpretations regarding fiber bridging, matrix densification, and ongoing geopolymerization reactions remain speculative, primarily based on the macroscopic trends and literature analogies, and require further validation.

On this basis, the results of this study provide reliable reference data for the performance evaluation of LRS geopolymer under freeze–thaw cycles in a terrestrial environment and lay a preliminary foundation for the future development of lunar environmental-adaptive materials. To advance research in this field, future work can be extended in the following directions:

(1) Environmental adaptability deepening: Conduct freeze–thaw tests under vacuum and anhydrous conditions to progressively approximate the authentic lunar environment, thereby validating and refining the existing mechanistic models;
(2) Microscopic mechanism validation: Introduce techniques such as scanning electron microscopy and mercury intrusion porosimetry to systematically analyze fiber-matrix interfaces and pore structure evolution, providing direct evidence for macroscopic performance;
(3) Multi-factor coupling investigation: Further explore the synergistic effects of freeze–thaw cycles, vacuum environment, and low gravity on the long-term durability of fiber-reinforced LRS geopolymer, providing a scientific basis for the engineering design of future lunar in situ construction materials.

The advancement of the above research directions will help, based on existing achievements, to gradually establish a material performance evaluation system from ground-based validation to lunar surface application.

## Figures and Tables

**Table 2:** Physical properties of basalt fibers.

Monofilament Diameter	Density (g/cm^3^)	Elastic Modulus (GPa)	Tensile Strength (MPa)	Service Temperature	Bonding Temperature
10 μm	2.63~2.65	91~110	3000~4800	−269–650 °C	1050 °C

**Table 3:** Proportion of LRS geopolymer.

Number	Fiber Content (%)	Freeze–Thaw Cycles	Water/Binder Ratio	Modulus	Na_2_O (%)	LRS (g)	Na_2_SiO_3_ (g)
G-0	0	0	0.456	3.3	8.3	100	100
GF-0	0.1	0				100	100
G-3	0	3				100	100
GF-3	0.1	3				100	100
G-6	0	6				100	100
GF-6	0.1	6				100	100
G-10	0	10				100	100
GF-10	0.1	10				100	100

Note: The modulus refers to the SiO_2_/Na_2_O molar ratio of sodium silicate (which is 3.3 in this study). Both the fiber content and the Na_2_O content are expressed as percentages by mass of LRS (% by mass of LRS), with the fiber content being 0.1%.

**Table 6:** Reference value of peak strain.

Number of Freeze–Thaw Cycles	0	3	6	10
Unreinforced	0.025	0.014	0.024	0.018
Basalt fiber-reinforced	0.017	0.027	0.020	0.021

## Data Availability

The original contributions presented in this study are included in the article. Further inquiries can be directed to the corresponding author.
